# Specific aeroallergen sensitization associated with current rhinitis among adults in southwestern Saudi Arabia

**DOI:** 10.1097/MD.0000000000030384

**Published:** 2022-09-02

**Authors:** Badr Rashed Al-Ghamdi, Fakhreldin Mohamed Omer, Nabil J. Awadalla, Ahmed A. Mahfouz

**Affiliations:** a Department of Internal Medicine, College of Medicine, King Khalid University, Abha, Saudi Arabia; b Department of Clinical Microbiology, College of Medicine, King Khalid University, Abha, Saudi Arabia; c Department of Family and Community Medicine, College of Medicine, King Khalid University, Abha, Saudi Arabia.

**Keywords:** adults, aeroallergens, current rhinitis, southwestern Saudi Arabia

## Abstract

Data about allergic sensitization to rhinitis among adults are limited. The objectives were to explore the prevalence of current rhinitis (CR) and associated specific allergen sensitizations in southwestern Saudi Arabia. A cross-sectional study was conducted on 969 adults in southwestern regions of Saudi Arabia, namely Aseer, Jazan, and Al Baha. From each region, 5 primary health care centers were chosen. The validated Arabic Version of the International Study of Asthma and Allergies in Childhood questionnaire was used. Total immunoglobulin E (IgE) enzyme-linked immunosorbent assay, cytokine enzyme-linked immunosorbent assay (interleukin [IL]-4, IL-10, IL-13, and interferon-γ), aeroallergen-specific IgE immunoassay (a panel of 30 common aeroallergens; 9 indoor and 21 outdoors), and eosinophilic count were assessed. A prevalence of CR of 35.8% (95% confidence interval: 32.8%–38.9%) was found. Regarding outdoor aeroallergens, *Mesquite*-positive IgE antibodies were higher among CR adults (odds ratio = 1.52, 95% confidence interval: 1.02–2.21) compared to those without CR. The same significant pattern was found with *Chenopodium*, *Ragweed*, *Pigweed*, *Russian thistle*, *Bermuda grass*, *Timothy grass*, and *Rye*. All indoor aeroallergens were not significantly associated with CR. Total IgE and eosinophil count were significantly higher among adults with CR. In conclusion, CR in southwestern regions of Saudi Arabia is common and of significant public health importance. Aeroallergens that associate with adult sensitization to CR tend to be of the outdoor variety particularly the herbaceous grass and their pollens. The magnitude of CR and its association with exposure to outdoor aeroallergens should be taken into account by health policy decision makers, clinicians, and medical practitioners when diagnosing and treating related conditions.

## 1. Introduction

Rhinitis is a common global sickness that is connected with hypersensitivity of the nose tissues due to immunological inflammation developing from numerous kinds of allergens. Its symptoms are coupled with itchy and runny nose, nasal block as well as sneezing which give away with or without using medication.^[1,2]^ It is a worldwide disease affecting more than 20% of persons around the world^[3]^ and its increasing prevalence was detected in many western and developing countries as well.^[4]^ A recent study conducted among general Saudi adult population during 2017 found an overall prevalence of rhinitis of 32.8%.^[5]^ Current rhinitis (CR) is an inflammatory type I hypersensitivity condition which, like other type I allergies is immunoglobulin E-mediated.^[6]^ High levels of eosinophils have been linked to CR, and nasal smear assessment for eosinophils may be a possible useful test to expect persistent or recurrent CR.^[7]^ Currently, data about allergic sensitization to CR among adults are limited and seem to require more studies.^[8]^

Although the cost in health and human suffering of CR is appreciated world-wide and in parts of the Middle East,^[9,10]^ its exact burden in Saudi Arabia in general and in southwestern regions in particular has not been systematically studied. In the past, as well as in the present, there are reports of the prevalence of CR in scattered localities within the country.^[11–14]^ Most of these studies targeted certain areas and, apart from a few, concentrated mainly on children of school age.^[15]^ Some of these studies revealed some information regarding the nature of local allergens linked to rhinitis sensitization plus an intricate association with asthma incidence.^[16,17]^ In general, studies on CR in adults, or late adolescence, have not been considered as a separate entity, but always in the context of asthma surveys.^[18]^ At present, there is a need for more systematic surveying of the status of CR and associated allergens at the subnational level within the Kingdom of Saudi Arabia. Such information will help with diagnosis, treatment as well as instituting suitable and well-targeted health policies similar to those in other parts of the world.^[19,20]^

This research aimed to explore the prevalence of CR and associated specific allergen sensitizations in southwestern Saudi Arabia.

## 2. Methods

### 2.1. Design

A cross-sectional study was conducted on a representative sample of adults in the 3 geographical areas of southwestern regions of Saudi Arabia, namely Aseer, Jazan, and Al Baha.

### 2.2. Target population and sample size

Health care delivery in Saudi Arabia is provided mainly through a wide-spread network of Primary Health Care Centers (PHCCs). Adults visiting the chosen PHCCs, for any purpose, were the target population. An adult was defined as any person aged >20 years old. Considering an anticipated conservative proportion of CR of 28.8% according to a study done in the Riyadh region of Saudi Arabia,^[21]^ absolute precision of 3% at 95% confidence interval (CI), the calculated sample size^[22]^ needed for the research was 876 persons.

### 2.3. Sampling technique

From each of the 3 regions, 5 PHCCs were randomly chosen, taking into consideration rural-urban differences and high altitude-sea level locations. Regular visits to the selected centers during 2018 were arranged by the study field teams. During the visits, adult males and females attending the selected PHCCs for any reason were invited to participate in the study (>95% response rate) and included consecutively till the required sample size was achieved. They included apparently healthy persons attending the centers accompanying other family members for services such as antenatal care, immunization, well baby clinics, dental care, chronic disease clinics, first aids, and premarital counseling services. A signed written informed consent was obtained from each subject before inclusion in the study. The sample participants were distributed among the different PHCCs using the proportionate sample size method.

### 2.4. Data collection sheet

The International Study of Asthma and Allergies in Childhood (ISAAC) questionnaire was used. Compared to the European Community Respiratory Health Survey for adults, it was found to be adequately valid. We used the Arabic validated version of the ISAAC.^[23,24]^ In the present study questions related to CR were used. “Having a problem with sneezing, or a runny, or blocked nose when DID NOT having a cold or the flu in the past 12 months,” as stated in the ISAAC, was applied to identify CR. CR symptoms were self-reported by individuals. Written informed consent was taken from each participant.

### 2.5. Immunological studies

Blood samples were obtained from all participants by venipuncture and processed to obtain sera. The following tests were performed: total immunoglobulin E (IgE) enzyme-linked immunosorbent assay, cytokine enzyme-linked immunosorbent assay (interleukin [IL]-4, IL-10, IL-13, and interferon-γ), aeroallergen-specific IgE immunoassay, and eosinophilic count. Individual tests were done following the manufacturer’s recommendations.

For specific IgE assays, a panel of 30 common aeroallergens (9 indoor and 21 outdoors) was selected from a review of the literature. The aeroallergens in the panel selected were mostly inhalant indoor household and outdoor allergens. The common aeroallergen panel included house dust mites, molds, and animal sources of allergens. Outdoor aeroallergens consisted of grass, trees bark, pollens, and fungus-derived spores. These allergens were selected by the investigator team based on previous research efforts to identify common allergens that are endogenous and are part of the natural fauna and flora of the study area. Previous local studies in the KSA have shown a link between the presence of such aeroallergens and the assessment of sensitization in other atopic conditions.^[17,25]^

### 2.6. Ethical considerations

The study proposal was presented to and officially approved by the Research Ethics Committee at King Khalid University (REC # 2014-06-11). Written informed consent was taken from study participants. Confidentiality and privacy of participants were guaranteed.

### 2.7. Data analysis

Statistical software IBM SPSS Statistics for Windows, version 22 (IBM Corp., Armonk, NY) was used for data analysis. Frequencies, arithmetic mean, standard deviations, and crude odds ratios (OR) and corresponding 95% CIs were used to present results.

## 3. Results

### 3.1. Description of the study sample

The present study included 969 adults comprising 718 men (74.1%) and 251 women (25.9%). Their age ranged from 20 to 85 years with an average of 37.63 ± 12.26 years.

### 3.2. Prevalence of adults with CR

Results revealed that 347 persons stated having CR in the past 12 months with a prevalence of 35.8% (95% CI: 32.8%–38.9%). Among males, the prevalence of CR in the past 12 months was 36.2% (260) than 34.7% (87) among females. The difference was not statistically significant (*P* = .702).

### 3.3. IgE antibodies specific to aeroallergens connected to adult CR

The frequency of positive outdoor aeroallergens-specific IgE antibodies among individuals with CR and those without CR was found in Table 1. The prevalence of *Mesquite*-positive IgE antibodies was higher among CR adults (14.6%) than in adults without CR (10.1%). The difference was found to be statistically significant (OR = 1.52, 95% CI: 1.02–2.21). The following outdoor aeroallergens showed the same significant pattern with regard to specific IgE levels. These were *Chenopodium*, *Ragweed*, *Pigweed*, *Russian thistle*, *Bermuda grass*, *Timothy grass*, and *Rye.*

**Table 1:** Distribution of positive IgE antibodies specific to outdoor aeroallergens among the study sample of adults.

Aeroallergens	Number of cases with positive specific IgE antibodies		cOR (95% CI)
	Non-current rhinitis, n/N (%)	Current rhinitis, n/N (%)	
**Outdoor**			
Acacia	35/622 (5.6%)	23/347 (6.6%)	1.19 (0.69–2.05)
Cypress	6/621 (1.0%)	2/349 (0.6%)	0.59 (0.12–2.94)
Juniper	15/622 (2.4%)	11/349 (3.2%)	1.32 (0.59–2.90)
Mesquite	63/622 (10.1%)	51/349 (14.6%)	**1.52 (1.02–2.21**)*
Date Palm	87/622 (14.0%)	62/349 (17.8%)	1.33 (0.93–1.90)
Willow	34/621 (5.5%)	24/349 (6.9%)	1.27 (0.74–2.19)
Alfalfa	38/621 (6.1%)	33/349 (9.5%)	1.60 (0.98–2.60)
Chenopodium	71/621 (11.4%)	58/349 (16.6%)	**1.54 (1.06–2.24**)*
Mugwort	24/622 (3.9%)	17/349 (4.9%)	1.27 (0.67–2.41)
Goat epithelium	75/622 (12.1%)	56/349 (16.0%)	1.40 (0.96–2.02)
Ragweed	95/622 (15.3%)	75/349 (21.5%)	**1.52 (1.08–2.12**)*
Pigweed	55/623 (8.8%)	46/349 (13.2%)	**1.57 (1.03–2.37**)*
Russian thistle	73/622 (11.7%)	57/346 (16.5%)	**1.48 (1.02–2.16**)*
Bermuda grass	75/622 (12.1%)	63/349 (18.1%)	**1.61 (1.12–2.31**)*
Timothy grass	67/620 (10.8%)	57/348 (16.4%)	**1.62 (1.10–2.37**)*
Rye	10/622 (1.6%)	15/349 (4.3%)	**2.75 (1.22–6.19**)*
Aspergillus mix	3/622 (0.5%)	1/348 (0.3%)	0.59 (0.06–5.74)
Feather mix	0/622 (0%)	0/348 (0%)	–
Horse	1/623 (0%)	0/348 (0%)	–
Camel	1/623 (0%)	0/348 (0%)	–
Sheep	0/621 (0%)	0/348 (0%)	–

cOR = crude odds ratio, IgE = immunoglobulin E, n = number positive, N = number tested.

* Significant (*P* ≤ .05).

Table 2 shows the frequency of positive indoor aeroallergens-specific IgE antibodies among individuals with CR and those without CR. All indoor aeroallergens were not significantly different among CR adults compared to those without CR.

**Table 2:** Distribution of positive IgE antibodies specific to indoor aeroallergens among the study sample of adults.

Aeroallergens	Number of cases with positive specific IgE antibodies		cOR (95% CI)
	Non-current rhinitis, n/N (%)	Current rhinitis, n/N (%)	
**INDOOR**			
Alternaria alternate	2/622 (0.3%)	4/349 (1.1%)	3.59 (0.65–19.72)
Cladosporium	4/622 (0.6%)	3/348 (0.9%)	1.34 (0.30-6.04)
Penicillium mix	0/623 (0%)	0/347 (0%)	–
Yeast mix	16/623 (2.6%)	12/349 (3.4%)	1.35 (0.63–2.90)
Cat epithelium	17/623 (2.7%)	10/348 (2.9%)	1.05 (0.47–2.33)
Cockroach mix	29/621 (4.7%)	13/348 (3.7%)	0.79 (0.41–1.54)
Storage mite	11/623 (1.8%)	12/347 (3.5%)	1.99 (0.87–4.56)
*Dermatophagoides petronyssinus*	89/623 (14.3%)	58/347 (16.7%)	1.20 (0.84–1.72)
*Dermatophagoides farina*	58/621 (9.3%)	43/346 (12.4%)	1.40 (0.91–2.09)

cOR = crude odds ratio, IgE = immunoglobulin E, n = number positive, N = number tested.

*Significant (*P* ≤ .05).

### 3.4. Total IgE positivity, cytokine levels, and eosinophil count associated with adult CR

Regarding positivity of total IgE, eosinophil count, and cytokines, the study showed that the level of total IgE (>100 mmol/L) was significantly higher (OR = 1.34, 95% CI: 1.04–1.76) among adults with CR (56.5%) compared to those without CR (49.0%). Similarly, eosinophil count (>150 cells/mm^3^) was significantly higher (OR = 1.75, 95% CI: 1.01–3.04) among adults with CR (62.4%) compared to those free of CR (48.6%). On the other hand, levels of cytokines studied were not found to be statistically significantly different.

## 4. Discussion

Globally, the prevalence of CR is rising. World Health Organization report indicates that about 40% of the general population may suffer from allergic diseases, including CR.^[26]^ The present study in the southwestern part of Saudi Arabia (Aseer, Jazan, and Al Baha regions) revealed a prevalence of CR among 35.8% (95% CI: 32.8%–38.9%). A recent study in Denmark reported a rising trend in the prevalence of allergic respiratory diseases over the preceding decades.^[27]^ This increasing trend may be attributed to environmental factors, including the increasing level of air pollution, climatic changes, and heat trapping.^[28]^ The prevalence of CR among adults in Europe and the USA ranges from 10% to >30%.^[29,30]^ In Asia, this allergic disease influences a large population sector, varying from 27% in South Korea^[31]^ to 53% in Malaysia.^[32]^

In Saudi Arabia, studies showed that the prevalence of CR was varying by geographical location. It was 24.7% in the western region,^[33]^ 34% in different regions,^[34]^ and 51% in Hail.^[15]^ A recent study conducted among general Saudi adult population during 2017 found an overall prevalence of rhinitis of 32.8%.^[5]^ The differences may be attributed to variations in the criteria of the study population, study tools, and the extent of environmental exposures.^[31]^ Saudi Arabia is affected by frequent sandstorms in all seasons. Sandstorms may carriage numerous types of allergens and dirt particles, which can initiate or exacerbate respiratory allergic respiratory diseases, including CR.^[35]^ Another contributing factor for the relatively high CR prevalence in Saudi Arabia may be related to the rapidly changing lifestyle and environmental aspects, including urban living, smoking, and air pollution.^[36]^

This study’s main finding is that aeroallergens associated with adult sensitization to allergic rhinitis tend to be of the outdoor variety, mainly the herbaceous grass and their pollens. They were Mesquite, *Chenopodium*, *Ragweed*, *Russian thistle*, and *Bermuda grass*. Traditionally these allergens tended to associate with seasonal rather than perennial or occupational sensitization. Studies have usually shown that seasonal sensitization to allergic rhinitis was associated with many outdoor allergens.^[37]^ In most of these studies, and like in our study, pollens were an important culprit. As a result of exposure to outdoor aeroallergens, CR should be taken into account by clinicians and medical practitioners when diagnosing and treating.^[38]^

Although exposure to indoor allergens like dust mites and molds could associate with rhinitis symptoms,^[39]^ this was not evident in our study. In children in the Najran area, Saudi Arabia, the risk factor for asthma and CR was associated with exposure to grass pollens.^[40]^ It is important to note that this is not unique to this part of the world as it has been reported from other parts of the world (Germany).^[41]^ In a study on environmental triggers for adults’ allergic rhinitis in neighboring United Arab Emirates, 7% of the studied population showed sensitization to CR due to exposure to grass pollens, house dust mites, and proximity to animals.^[42]^ In a large-scale nationwide study in Saudi Arabia, using a self-report electronic survey distributed via social media networks in 2018, the major environmental triggers were found to be dust (74%) followed by pollens (17%).^[43]^

Our study has shown a significant association between high serum total IgE levels and eosinophil counts, and CR sensitization. While this type of association has been reported to various respiratory allergy,^[44]^ the exact association with CR is not sufficiently clear since non-allergic rhinitis is a confounding factor.^[45]^ A role for serum eosinophil counts and total and specific IgE in guiding treatment has been suggested.^[44,46]^

However, this study had some limitations. First, the data were obtained using a self-administered questionnaire that may be affected by recall bias. Similarly, the preponderance of males in the sample selected is another limitation. The study relied entirely on healthy persons attending the centers for any reasons. Culturally, men are usually accompanying their family members during the visit.

## 5. Conclusion

The present study reported a high prevalence rate of CR in Saudi Arabia in southwestern regions, revealing a significant public health problem. The study identified aeroallergens that associate with adult sensitization to allergic rhinitis which tend to be of the outdoor variety particularly the herbaceous grass and their pollens including *Mesquite*, *Chenopodium*, *Ragweed*, *Russian thistle*, and *Bermuda grass*. The magnitude of CR and its association with exposure to outdoor aeroallergens should be taken into account by health policy decision-makers, clinicians, and medical practitioners when diagnosing and treating related conditions. On the other hand, the small size of indoor allergens may be behind the absence of difference between those with CR versus no rhinitis

## Author contributions

**Conceptualization:** Badr Rashed Al-Ghamdi, Fakhreldin Mohamed Omer

**Data curation:** Fakhreldin Mohamed Omer, Nabil J. Awadallac, Ahmed A. Mahfouz

**Formal analysis:** Ahmed A. Mahfouz, Nabil J. Awadalla

**Investigation:** Fakhreldin Mohamed Omer

**Methodology:** Badr Rashed Al-Ghamdi, Fakhreldin Mohamed Omer, Ahmed A. Mahfouz, Nabil J. Awadalla

**Resources:** Badr Rashed Al-Ghamdi

**Supervision:** Badr Rashed Al-Ghamdi

**Writing – original draft**: Badr Rashed Al-Ghamdi, Fakhreldin Mohamed Omer, Ahmed A. Mahfouz, Nabil J. Awadalla

**Writing – review & editing:** Badr Rashed Al-Ghamdi, Fakhreldin Mohamed Omer, Ahmed A. Mahfouz, Nabil J. Awadalla

**Conceptualization:** Ahmed A. Mahfouz, Badr Rashed Al-Ghamdi, Fakhreldin Mohamed Omer, Nabil J. Awadalla.

**Formal analysis:** Ahmed A. Mahfouz, Nabil J. Awadalla.

**Funding acquisition:** Badr Rashed Al-Ghamdi.

**Investigation:** Badr Rashed Al-Ghamdi, Fakhreldin Mohamed Omer.

**Methodology:** Ahmed A. Mahfouz, Badr Rashed Al-Ghamdi, Fakhreldin Mohamed Omer, Nabil J. Awadalla.

**Project administration:** Badr Rashed Al-Ghamdi.

**Writing – original draft:** Ahmed A. Mahfouz, Badr Rashed Al-Ghamdi, Fakhreldin Mohamed Omer, Nabil J. Awadalla.

**Writing – review & editing:** Ahmed A. Mahfouz, Badr Rashed Al-Ghamdi, Fakhreldin Mohamed Omer, Nabil J. Awadalla.
